# Machine learning-based determination of sex-related bladder cancer biomarkers

**DOI:** 10.3389/fbinf.2026.1794098

**Published:** 2026-04-29

**Authors:** Joseph R. Pizzi, Image Adhikari, Prakyat Prakash, Hangchuan Shi, Hiroshi Miyamoto, Feng Cui

**Affiliations:** 1 Thomas H. Gosnell School of Life Sciences, College of Science, Rochester Institute of Technology, Rochester, NY, United States; 2 Department of Computer Science, Golisano College of Computing and Information Sciences, Rochester Institute of Technology, Rochester, NY, United States; 3 Department of Pathology and Laboratory Medicine, University of Rochester Medical Center, Rochester, NY, United States; 4 Department of Urology, University of Rochester Medical Center, Rochester, NY, United States

**Keywords:** bladder cancer, feature selection, gene expression, machine learning, sex dimorphism, sex hormones

## Abstract

**Introduction:**

Bladder cancer exhibits sex-specific behavior, occurring more frequently in males but progressing to advanced stages more commonly in females. The activation of sex hormone receptors may explain these differences, but the exact genetic drivers remain poorly understood. Furthermore, current bladder cancer biomarkers have inconsistent sensitivities and specificities in practice, making early diagnosis a challenge.

**Methods:**

This study approaches bladder cancer biomarker discovery through machine learning techniques on gender and disease-stratified RNA-seq data. Training sets limited to differentially expressed genes were subjected to four different feature selection methods: differential gene expression analysis adjusted p-value, recursive feature elimination with support vector machine, logistic regression, and an optimized random forest procedure. Gene panels were compared and aggregated across selection strategies and cross-validation folds to identify robust biomarkers for sex-specific bladder cancer development and progression.

**Results:**

When applied to unseen datasets and limited to 50 genes or less, male and female-specific panels achieved areas under the receiver operating characteristic curve of 0.932 and 0.914, respectively, in distinguishing bladder cancer samples from non-tumor controls. In terms of enriched pathways, the male panel was associated with cell interactions and altered PI3K-AKT signaling, while the female panel was more closely connected to extracellular matrix reorganization. The panel differentiating male and female tumors had a poorer performance on external datasets compared to the sex-specific analyses, but still contained relevant genes.

**Discussion:**

Genes such as PRAC1 and PCDH11Y were identified as high-impact predictors related to sex hormones or chromosomes for male tumor development. In the female-specific panel, genes related to aberrant androgen signaling across tumor types like androgen receptor, PLXNA1, USP54, and PMEPA1 were influential. These results offer potential targets for further in vivo/vitro experimentation and provide a framework for constructing high-performance gene panels related to sex-specific bladder cancer biology.

## Introduction

Bladder cancer (BCa) is the sixth most common cancer in the United States, representing 4.2% of all cancer cases (SEER, 2025). The 5-year relative survival rate is estimated at 79.0%, but individual prognosis depends heavily on the stage and aggressiveness of the tumor. However, identifying BCa early in development can be difficult, with most symptomatic individuals presenting with gross hematuria, a malady common in other diseases like urinary tract and kidney infections (Jakus et al., 2023). The current diagnostic standard is cystoscopy, yet this method relies on human judgment and can miss carcinoma *in situ* without further imaging (Lopez-Beltran et al., 2024). Cystoscopy is often preceded by urine cytology to identify abnormal cells characteristic of high-grade tumors but this technique can prove ineffective for lower-grade cases. To mitigate risk while prioritizing prompt detection, the FDA has explored and approved less-invasive molecular biomarkers for use in the clinic. Unfortunately, their performance is unreliable, with nuclear matrix protein 22 and bladder tumor antigen tests exhibiting highly variable sensitivities and specificities (Flores Monar et al., 2023). While they offer an improvement upon cytology in terms of sensitivity, current BCa biomarkers still suffer from high false-positive rates.

Gender-specific differences in frequency and pathogenesis add another layer of complexity to the task of better diagnosing and treating BCa. Across all races, the incidence rate of BCa in males is approximately 3.3 times what is observed in females (Siegel et al., 2025). Only part of this phenomenon can be attributed to a higher percentage of men being smokers, with a 2020 study finding that men had a prevalence of 32.6% while women stood at 6.5% (Dai et al., 2022). Despite the stark difference in frequency, females tend to have more aggressive forms of BCa upon diagnosis. A recent French study found that only 8.9% of women with muscle-invasive BCa had a history of the non-invasive variant, while men did at a higher rate of 26% (Poli et al., 2025). Systematic biases in the healthcare system can partially explain this. Women presenting with hematuria are typically less likely to be referred to a urologist, with a 29% referral rate compared to 45% for men in NCI’s Southern Community Cohort Study (Ark et al., 2017). However, females still tend to have lower survival rates compared to men, even after correcting for confounding variables like tumor stage and demographics (Radkiewicz et al., 2020).

These findings suggest that there are molecular mechanisms driving gender-specific differences in BCa outcomes in addition to population characteristics. Differences in androgen receptor (AR) signaling have been explored as a critical factor in BCa sex dimorphism (Li et al., 2017). In canonical signaling, the binding of androgens like testosterone and dihydrotestosterone to AR forms a dimer complex that reaches the nucleus and binds androgen response elements to shift transcriptional patterns (Kolyvas et al., 2022). In bladder tissue, AR signaling has been linked to urine storage, urinary tract function, nerve functions, and urothelium musculature/thickness (Li et al., 2017). Preliminary research suggests that aberrant AR activity may influence BCa development/progression through potential downstream targets like *ADGRL3*, *ELK1*, and *FOXO1* (Goto et al., 2024; Ide et al., 2020; Elahi Najafi et al., 2025). Individuals with male reproductive organs tend to have higher levels of androgens in their blood circulation than females, which could contribute to the increased prevalence of the disease in men (Doshi et al., 2023). On the other hand, estrogen receptor (ER) activity in females may contribute to the observed increased tumor aggressiveness compared to males. In traditional ER signaling, similarly to the mechanism of AR activity, estrogen-bound ERα and ERβ act as transcription factors (Fuentes and Silveyra, 2019). Reviews of current BCa research have revealed the importance of ERβ, with ERβ positivity ranging from 27%–100% in urothelial tumors across various publications compared to 0%–38% exhibiting ERα positivity (Goto and Miyamoto, 2021). Potential downstream effectors of ERβ in BCa cells have been identified. For example, ERβ activation was demonstrated to reduce *GULP1* expression to elicit cisplatin resistance in BCa cell lines (Tatenuma et al., 2024). Outside of steroid hormone signaling, sex chromosome differences may contribute to BCa sex dimorphism as well. For example, the *KDM6A* gene is a tumor suppressor located on the X chromosome that can escape inactivation, leading to enhanced protection in females due to having an extra copy (Koti et al., 2020). Specifically, *KDM6A*-lacking organoids implanted in mice had increased expression of basal genes and decreased expression of luminal genes (Qiu et al., 2023). Overall, the genetic and cellular mechanisms underlying higher BCa prevalence in men but more aggressive progression in women are still unclear and require further study.

The lack of robust, sensitive biomarkers in tandem with established gender differences in disease development calls for the discovery of new BCa biomarkers. This study approached this problem using machine learning, where expression data is split into training and testing sets for a classification task. Here, three classification tasks were handled with machine learning: male/female tumor *versus* non-tumor and male *versus* female tumors based on publicly available tissue-level high-throughput sequencing data. First, differential gene expression analysis (DGEA) was applied as a filter-based feature selection method to reduce the sparsity of the gene expression data and highlight differentially expressed genes (DEGs) related to sex and tumorigenesis. Then, four different feature selection approaches - DGEA adjusted p-value, an optimized random forest (RF) procedure, recursive feature elimination with support vector machine (SVM-RFE), and logistic regression - were applied to task-specific DEGs to isolate high-impact biomarkers. Afterward, the rankings for each approach were aggregated across fold schemes into one synthesized gene panel for each classification task using robust rank aggregation (RRA). The effectiveness of aggregated and single-method panels was evaluated on external testing sets using multiple machine learning models based on a combined metric that takes class imbalance into account. Pathway enrichment and protein-protein interaction (PPI) network analysis were also carried out to reveal the prominent molecular mechanisms within each aggregated panel. In the end, robust, biologically relevant, and sex-related biomarkers for BCa development and progression that generalize well to unseen data were identified.

## Materials and methods

### Data stratification and cross-validation scheme

**FIGURE 1:**
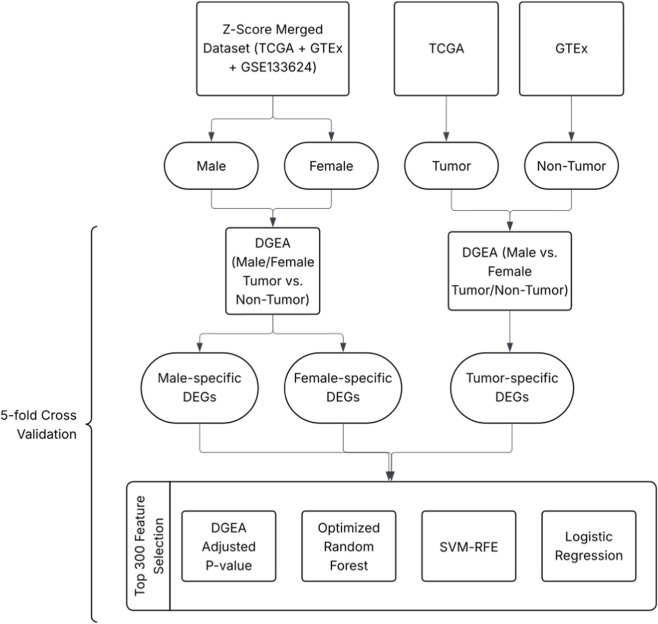
Flowchart of DGEA and feature selection workflow. DEGs specific to differentiating non-tumor males and females are only used to correct for tumor-specific DEGs and do not undergo feature selection.

To elucidate biomarkers associated with gender-specific development and gender-related progression, two analyses were conducted in parallel: one stratified by gender and another stratified by disease status. The gender-stratified study contrasted the expression profiles of tumor tissue *versus* non-tumor tissue for each gender, while the disease status-stratified study focused on differences between males and females in both diseased and non-tumor tissue. These two stratifications created three core classification tasks: male tumor *versus* male non-tumor, female tumor *versus* female non-tumor, and male tumor *versus* female tumor. The gene feature selection pipeline was set up with 5-fold cross-validation, where the training data for each task was initially divided into 5 equal partitions. This split was stratified, meaning that the class ratio of the whole dataset was maintained across each fold. Then, the entire pipeline (Figure 1) was executed 5 times, with a different partition acting as the internal validation set in each iteration, while the remaining four folds were used for DGEA and feature selection. A cross-validated approach was employed for all three classification tasks to ensure the selected biomarkers were robust. The training data for the sex-specific development and sex-related progression analyses differed slightly. In classifying male tumors *versus* female tumors, the TCGA-BLCA dataset had a reasonable number of samples for each group: 267 male tumors and 102 female tumors after removing stage I, metastasized, and low-grade samples from consideration (Table 1). However, under 5% of the entire TCGA consisted of non-tumor controls, making the sex-stratified tumor *versus* non-tumor task impossible to execute. In response, a merged cohort was created to combat this extreme class imbalance. TCGA-BLCA, GTEx, and GSE133624 were independently processed and z-scored before being concatenated together on common genes in the GRCh38.p13 reference genome.

**TABLE 1:** Class distribution of each dataset. TCGA, GTEx, and GSE133624 were independently z-scored, then merged to constitute the training set for the male/female non-tumor *versus* tumor analyses. When used alone, the TCGA data was corrected to remove metastasized and lower staged samples.

Dataset	Use	Total	Male tumor	Female tumor	Male non-tumor	Female non-tumor
TCGA	Training	431	304	108	10	9
GTEx	Training	77	0	0	48	29
GSE133624	Training	65	32	4	25	4
GSE236932	External	58	27	8	15	8
GSE188715	External	70	46	11	11	2

RNA-seq data from the TCGA-BLCA project were downloaded from the Genomic Data Commons as raw counts. In addition to their gene expressions, clinical information (gender, tumor status, staging, *etc.*) for each patient was extracted *via* their patient ID. For the male tumor *versus* female tumor task, Stage I, low-grade, and metastasized tumors were excluded to ensure gender-related expression differences were not confounded. The male/female tumor vs. non-tumor analyses also incorporated GTEx and NCBI GEO data into this TCGA cohort. Non-tumor bladder tissue raw counts and clinical annotations from the GTEx V10 release were downloaded from the GTEx portal. GSE133624, the final dataset for the merged data pool containing both BCa and paired non-tumor tissue samples, was downloaded from the NCBI GEO as raw counts. For all datasets, Ensembl IDs were used to represent each gene to ensure a common nomenclature before merging. Counts were summed for IDs with multiple entries to avoid duplicate features.

### Dataset merging and differential gene expression analysis

To create the merged training dataset, all three cohorts were concatenated together on common Ensembl IDs. All genes not appearing across all datasets were excluded from consideration at this point. In the merged data matrix, the original dataset, gender, and disease status for each patient were retained. Afterward, the merged dataset was stratified into male and female subsets, while the TCGA and GTEx datasets acted as the tumor and non-tumor subsets, respectively. Each subset was then split into 5 folds as part of a cross-validation scheme. In this, each fold became the internal validation set while the remaining 4 folds were used for DGEA and feature selection for 5 iterations of the analysis in total for each classification task. The non-tumor subset was an exception to this, as machine learning classification of non-tumor males *versus* females was not deemed useful. Thus, DGEA for the GTEx data was done outside of the cross-validation scheme to identify DEGs differentiating non-tumor males and females.

All training sets corresponding to each stratified subset were then subjected to DGEA with DESeq2 (Love et al., 2014). In the case of the merged training data, the original dataset (batch) was added as a model covariate such that expression differences due to the condition of interest (disease status, gender) were not masked. DEGs between conditions were then selected at a threshold of adjusted p-value <0.05 and absolute log2 fold change >1.0. Ensembl IDs were then converted to gene symbols using the GRCh38.p13 reference genome.

### Data normalization

With DEGs for each stratified subset determined, the merged dataset was first reconstructed. Similarly to how batch was added as a covariate in the DESeq2 model, differences in distributions between datasets had to be accounted for before training feature selection machine learning models. To achieve this, each of the three datasets was independently normalized before concatenation. First, raw counts were converted to transcripts per million (TPM) values to account for transcript length differences between genes using the GRCh38.p13 reference genome. All TPM values were then subjected to a log_2_ transformation with a pseudocount of 1 to account for genes with no expression. Afterward, each dataset was independently z-scored to bring the mean to 0 and the standard deviation to 1 for each gene. All three normalized datasets were concatenated to create the merged cohort for the male/female tumor *versus* non-tumor tissue analyses. The efficacy of this correction technique was visualized through Uniform Manifold Approximation and Projection (UMAP) on the data merged with and without prior independent normalization. As a point of comparison, ComBat-seq was also employed to create a combined cohort for the male/female tumor *versus* non-tumor tasks. In this, the three datasets were concatenated while storing the original dataset of each sample as metadata. Then, the sva R package was applied with tumor status and gender as covariates followed by a TPM conversion and log2 transformation of the batch-corrected counts. The z-score and ComBat-seq merged datasets were analyzed in parallel using the same methods. For the male *versus* female tumor analysis, the TCGA raw counts were simply converted to TPM values with the GRCh38.p13 reference genome and then subject to a log_2_ transformation with a pseudocount of 1.

After normalization, the data for all stratified subsets were limited to the previously-identified DEGs. The same 5-fold split applied to the counts data was applied to the normalized TPM training data, so DEGs remained consistent within each fold scheme. However, a key caveat is that DEGs were limited to those unique to each subset. In this, genes differentially expressed between non-tumor and tumor samples for both genders were isolated. Afterward, DEGs with the same direction of regulation for both genders were removed from consideration. For the disease status-stratified analysis, genes differentially expressed between male and female samples for both tumor and non-tumor tissue were excluded from consideration unless oppositely regulated. The purpose of this step was to remove genes that have altered expression in BCa, regardless of gender, and genes with shifted expression between genders that may not be related to BCa. At this point, the data for the non-tumor cohort was deemed obsolete, as its sole purpose was to help remove irrelevant genes from the tumor cohort. As such, only the DEG-limited normalized training data from the male, female, and tumor cohorts moved on to the feature selection phase.

### Feature selection and aggregate panel construction

Processed training data for each stratified subset was subjected to four different feature selection strategies: DGEA adjusted p-value, optimized RF, SVM-RFE, and cross-validated logistic regression. Mori and colleagues executed 1,000 trials of their deep learning-based feature selection on pancreatic cancer gene expression data, tallying how many times each gene appeared in the top 10 and 50 feature importances (Mori et al., 2021). Olatunji and Cui applied this methodology to an RF model tallying the top 100 genes most important to distant metastasis at each iteration (Olatunji and Cui, 2023). The present approach built upon these implementations by tracking the top 300 genes and summing their RF feature importance values across all 1,000 iterations with different random seeds. Before the 1,000 iterations, the RF model was hyperparameter optimized with a stratified 5-fold cross-validated exhaustive grid search. The 300 genes with the highest feature importance sums were selected for evaluation for each classification task. For SVM-RFE, a linear kernel was used to utilize variable coefficients as a feature importance metric, and a stratified 5-fold exhaustive search was used to find the optimal value for the regularization parameter. Standardization was applied to each unique training fold within cross-validation rather than the entire inputted subset to avoid data leakage. While slightly stricter than other cancer biomarker studies, 0.1% of uninformative features were removed at each iteration of RFE until 300 remained. For the logistic regression model, a stratified 5-fold exhaustive search identified the optimal regularization parameter value for the L1 penalty, with saga as the solver based on validation performance. As with SVM-RFE, standardization was applied to each unique training fold within cross-validation rather than the entire inputted subset to avoid data leakage. The top 300 genes were selected based on the magnitude of their coefficients. The final feature selection approach extracted the top 300 DEGs by adjusted p-value with an absolute log_2_ fold change greater than 1.0. For all parameter searches, a composite metric taking the F1 score, the area under the receiver operator curve, and balanced accuracy into account was utilized to adjust for class imbalance (0.5*F1 + 0.3*AUROC + 0.2* balanced accuracy). The F1 score was weighted the most heavily due to its sensitivity to the true positive rate and established utility in imbalanced human health datasets (Diallo et al., 2025). AUROC is particularly relevant to diagnostic problems and is also robust to class imbalance, but is less established compared to the F1 score (Çorbacıoğlu and Aksel, 2023; Richardson et al., 2024). Finally, balanced accuracy was included with the smallest weight to represent a simpler measure of model performance. The inner 5-fold split for each hyperparameter grid search was executed on the training folds only. All machine learning models, feature selection approaches, and hyperparameter optimization searches were implemented with the scikit-learn package. The result of feature selection was four top 300-gene panels for each of the three cohorts within each cross-validation scheme.

**FIGURE 2:**
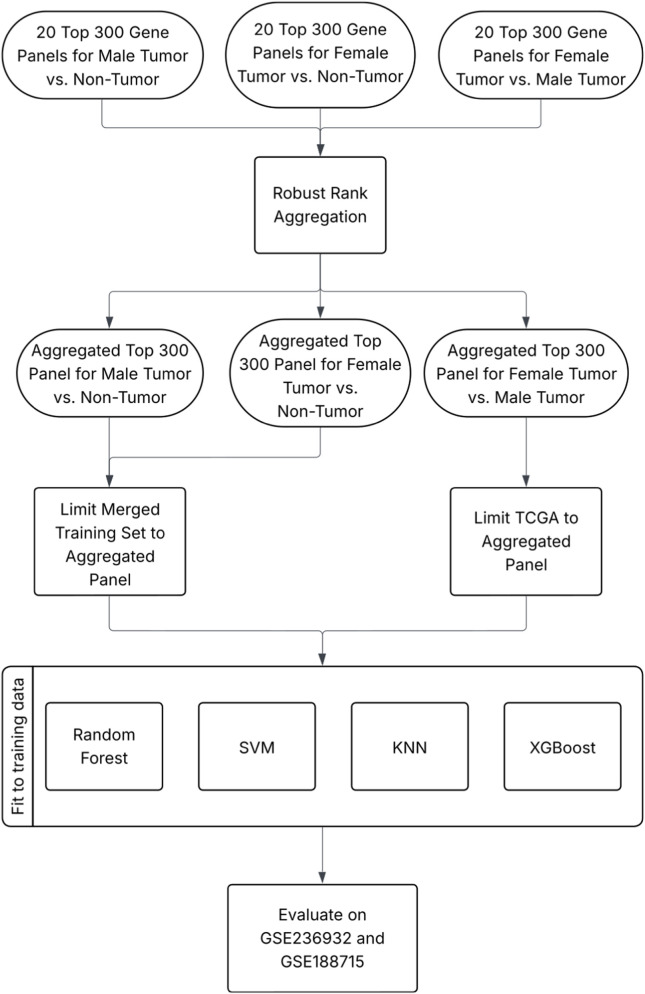
Flowchart of evaluation workflow. In the case of the merged training set for male/female tumor *versus* non-tumor, GSE236932 and GSE188715 were z-scored before scoring.

Across 5 fold schemes and four feature selection methods, 20 top 300-gene panels were generated for each classification task. All rankings within a task were combined into an aggregated top 300 panel using RRA to synthesize findings across all feature selection approaches and combinations of training folds (Kolde et al., 2012). This assigned a score to each gene, with lower scores being assigned to those highly ranked across all lists. To visualize the RRA scores, a negative log_10_ transformation was applied to the scores of the top genes and a bar graph was constructed with the matplotlib package. Consensus top 300 panels for each selection method were also produced, aggregating the five rankings produced by each fold scheme. The overlap in genes between the consensus panels for each feature selection approach was demonstrated by creating a Venn diagram for each classification task using the pyvenn package. The four cross-fold consensus panels and the aggregated ranking were then considered for validation on external datasets for each classification task (Figure 2).

### Gene panel evaluation

To evaluate each gene panel, four evaluation models were trained: RF, SVM with linear kernel, K-nearest neighbors (KNN) classifier, and XGBoost. XGBoost was outside of scikit-learn and required the installation of the xgboost module. For each, the classifier was introduced to the training data and then applied to the external testing sets, all limited to the gene panel of interest. The external cohorts consisted of RNA-seq NCBI GEO datasets GSE236932 and GSE188715, whose expressions were downloaded as TPM values calculated with GRCh38.p13. Genes with more than one entry had their expressions averaged, and the same log_2_ transformation with a pseudocount of 1 was applied, just as was done for the training data. For the male/female tumor *versus* non-tumor tasks, each external dataset was z-scored independently of the training data to bring them to the same scale. Ideally, the mean and standard deviation from the training set would be applied to the test data, but this was not possible due to standardization being done independently for each of the three training datasets prior to merging.

Each top 300 gene panel was further subsetted (top 300, 250, 200, *etc.*) to judge whether diminishing the feature count enhanced predictive ability. Before evaluating the classifiers on the test set, their hyperparameters were optimized on the training data using 5-fold cross-validation with the previously mentioned composite metric. For the distance-based models (SVM and KNN), the classifiers were pipelined with a standardization step to prevent data leakage during cross-validation. Due to their smaller hyperparameter spaces, exhaustive search was appropriate for SVM and KNN classifiers. For RF and XGBoost, a randomized grid search with 200 iterations and a Bayesian search implementation from scikit-optimize with 50 iterations were required to explore the much larger number of possible hyperparameter combinations. The performance of each subset on the internal and external testing sets was compared using accuracy, F1 score, AUROC, and the weighted composite metric. Additionally, the area under the precision recall curve (PR-AUC) was considered to include a class imbalance-sensitive performance metric outside of those used for the composite score. To obtain an uncertainty estimate for each metric, each external dataset was resampled with replacement to generate 500 bootstrap samples for evaluation. All performance metrics were computed on all bootstrap samples, and a 95% percentile bootstrap confidence interval was generated for each. ROC curves for each feature subset in each external dataset were produced using the RF, SVM, and XGBoost evaluation models, and those with the top AUROC scores were visualized together. Such a detailed evaluation of each top 300 gene panel identified the selection method and subset with the best classification performance for each task.

### Pathway enrichment and PPI network analysis

Pathway enrichment was carried out on the aggregated top 300 panel for each classification task using clusterProfiler from Bioconductor (Yu et al., 2012). Significant ontologies were identified as those having a q-value less than 0.05 and the KEGG pathways and GO biological process databases were utilized. However, at this threshold the male and female tumor *versus* non-tumor panels did not yield any significant hits, so the query was expanded to the top 500 genes for each task. Dot plots were constructed with the ggplot2 graphical interface to represent the top 10 pathways for each task/database combination.

PPI network analysis was performed by inputting the top 300 genes of each aggregated panel into the STRING database web interface to generate an edge-node graph of protein interactions. A TSV of node relationships was outputted and all gene products without edges or a combined score less than 0.4 were excluded from further analysis. All three networks were reconstructed by importing the STRING TSV into Cytoscape. Within Cytoscape, MCODE was used to identify significant gene modules while cytoHubba helped to execute topological analysis and isolate important nodes. For MCODE, a degree cutoff of 2, node score cutoff of 0.2, k-core of 2, and max depth of 100 were utilized with the haircut setting turned on. Significant clusters were defined as those having a score of three or greater. For cytoHubba, the top 10 genes in terms of maximal clique centrality were determined for each of the three networks and classified as hub genes. Pathway enrichment was performed for gene modules containing hub genes using the Enrichr web interface to obtain significant terms in the Reactome database (Xie et al., 2021). All pathways with a q-value of 0.05 or less were considered.

## Results

### Dataset merging

**FIGURE 3:**
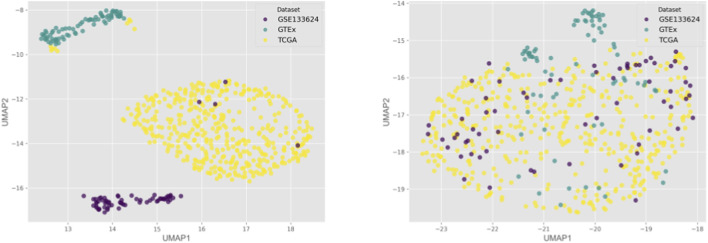
UMAP projections of merged dataset normalized TPM values before (left) and after (right) implementing independent z-scoring prior to merging.

In classifying male tumors *versus* female tumors, the TCGA-BLCA dataset had a reasonable number of samples for each group: 267 male tumors and 102 female tumors after removing stage I, metastasized, and low-grade samples from consideration (Table 1). However, under 5% of the entire TCGA dataset consisted of non-tumor controls, making the sex-stratified tumor *versus* non-tumor task nearly impossible to execute. In response, a merged cohort was created to combat this extreme class imbalance. TCGA-BLCA, GTEx, and GSE133624 were independently processed and z-scored before being concatenated together on common genes in the GRCh38.p13 reference genome. To visualize the effects of z-score merging, UMAP dimensionality reduction was performed on the normalized TPM data (Figure 3). When the three datasets were merged without independent z-scoring, samples clustered strictly based on their dataset. When z-scoring before concatenation, this clustering pattern was disrupted, and a more heterogeneous mixture of samples across datasets was observed. Data merged with ComBat-seq was also generated to act as a point of comparison in later analyses.

### Differential gene expression analysis and feature selection

**FIGURE 4:**
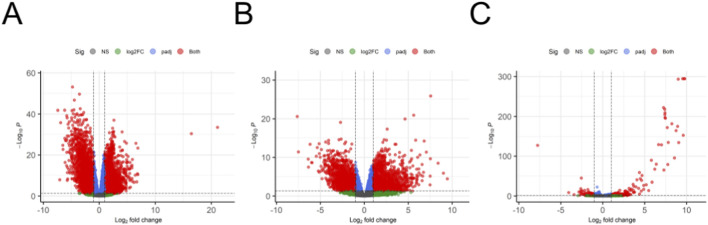
Volcano plots of DGEA for male tumor *versus* non-tumor **(A)**, female tumor *versus* non-tumor **(B)**, and male *versus* female tumors **(C)**.

Within each fold scheme, DGEA was carried out for male/female non-tumor *versus* tumor samples and male tumors *versus* female tumors. For all analyses, DEGs were defined as those with an adjusted p-value less than 0.05 and a log2 fold change greater than |1.0|. After comparing and excluding overlapping genes across cohorts, the result was three groups of DEGs: male-specific BCa development, female-specific BCa development, and sex-related BCa progression. This was also performed outside of the cross-validation scheme on the entire training dataset for each task and visualized with volcano plots (Figure 4). For the male-specific tumor development analysis, 2,800 DEGs were identified with 1,414 upregulated and 1,386 downregulated. In the female-specific development task, only 821 DEGs were significant with 525 upregulated and 296 downregulated. Finally, in comparing expressions between male and female samples, 328 DEGs were exclusive to diseased tissue with 223 upregulated and 105 downregulated.

**FIGURE 5:**
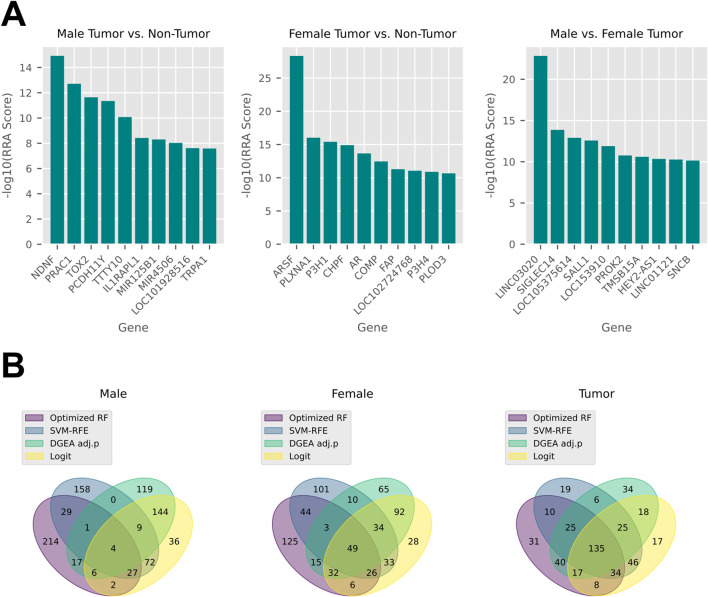
Aggregation of gene panels across fold schemes and selection methods. **(A)** Bar graphs depicting the top 10 genes in terms of RRA score in the cross-fold cross-method aggregated panel for each classification task. A negative log_10_ transformation was applied to each score. **(B)** Venn diagrams displaying the overlap in genes between the cross-fold consensus panels for each selection method in each classification task.

Each DEG pool was then subjected to feature selection using four different methods: optimized RF, SVM-RFE, logistic regression, and filtering *via* the DGEA adjusted p-value. Each strategy isolated the top 300 DEGs based on composite metric performance, which combined F1 score, the area under the receiver operating characteristic curve (AUROC), and balanced accuracy to account for class imbalance. Each task produced four top 300 gene panels in five different fold schemes to create 20 rankings in total. Through RRA, these were consolidated into an aggregated top 300 ranking, where genes placing highly across all 20 input panels were associated with a better score (Supplementary Tables S1–S3). The top 10 genes for each task in terms of RRA score were visualized as a bar graph by transforming their scores with a negative log transformation (Figure 5A). All cohorts had a clear top gene: NDNF, ARSF, and LINC03020 for male, female, and tumor, respectively, with scores leveling off towards the end of the top 20. The top 300 rankings for each selection method were also aggregated across fold schemes to create cross-fold consensus rankings, which were then compared to each other with a Venn diagram (Figure 5B). Regardless of the classification task, optimized RF and SVM-RFE tended to produce quite distinct panels, while logistic regression and DGEA adjusted p-value strategies had more genes in common with other methods. The male non-tumor *versus* tumor task had the least amount of agreement between the four selection methods, with only four genes being shared among all the consensus panels. On the other hand, different selection methods had much more of an overlap in the male tumor vs. female tumor task, with 135 genes shared between the aggregated panels for each.

### Internal validation

In each fold scheme, the testing fold acted as an internal validation set to determine how well the selected genes applied to each classification task on data similar to the training data. Four machine learning models, RF, SVM, KNN, and XGBoost, were hyperparameter optimized, fit on the four training folds, then tested on the final fold. Balanced accuracy, F1 score, AUROC, and the composite metric were averaged across all 5 folds for a given gene panel in all three classification tasks. The aggregate panels were not considered, as those were assembled outside of the cross-validation scheme. In the male tumor *versus* non-tumor classification task, the best performing panel was the optimized RF top 50 using XGBoost as the evaluation model with a composite metric of 0.988 (Supplementary Table S4). The following four best panels were also all derived from the optimized RF top 300, but some used RF as the evaluation model. In the female tumor *versus* non-tumor analysis, the top two panels were the SVM-RFE top 300 and 200 employed with XGBoost, demonstrating average composite scores of 0.965 and 0.963, respectively (Supplementary Table S5). Comparatively, the male tumor *versus* female tumor classification was much more difficult, with the logistic regression top 300 using RF evaluation having the best average composite metric of 0.734, but a more respectable average F1 score of 0.843 (Supplementary Table S6).

### External validation

**FIGURE 6:**
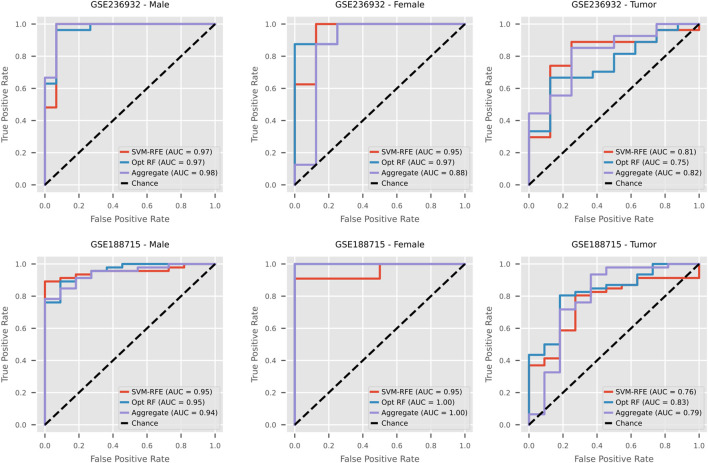
ROC curves of external validation performance. The panels with the highest AUROCs for the optimized RF, SVM-RFE, and aggregated rankings were chosen for visualization in each combination of classification task and external dataset. SVM-RFE and optimized RF were selected based on internal validation performance.

The same four models used for internal validation were re-trained on their respective full, unfolded training dataset then applied to two external datasets, GSE236932 and GSE188715. The cross-fold cross-method aggregated panel and the cross-fold panels for each selection method were considered in each classification task. First, ROC curves were constructed to compare between the aggregated and cross-fold individual selection method panels in each task/dataset combination (Figure 6). Due to their consistent high performance in internal validation, the SVM-RFE and Optimized RF panels with the highest AUROC were chosen to represent the individual selection methods. In the male tumor *versus* non-tumor classification, the AUROC of the best aggregated panel surpassed those of the single selection methods in the first external dataset and was comparable in the second. For female samples, the top aggregated panel was within 0.1 of the optimized RF selection AUROC and in GSE188715 obtained a perfect 1.0 due to sample size limitations. Finally, in differentiating male tumors from female tumors, the aggregated panel had the highest AUROC in GSE236932 and in GSE188715 surpassed the SVM-RFE panel. In GSE236932, SVM tended to be the best-suited evaluation model across all gene panels, but this shifted to XGBoost and RF in GSE188715. Across all datasets and panels, the highest AUROCs were obtained by panels with the top 100–300 genes. To examine the aggregated panels further, the performance metrics of each panel were averaged across both external datasets (Supplementary Tables S7–S9). The 95% percentile bootstrap confidence intervals for GSE236932 and GSE188715 were included as well. Then, the best aggregated panels with 50 or less genes were considered (Table 2). The aggregated male top 50 was able to achieve average AUROC and F1 scores of 0.923 (GSE236932: [0.782, 1.0], GSE188715: [0.843, 0.979]) and 0.917 (GSE236932: [0.796, 0.967], GSE188715: [0.866, 0.978]) (0.915 composite), respectively with RF. The average PR-AUC for this same panel was 0.95 (GSE236932: [0.775, 1.0], GSE188715: [0.958, 0.996]). In the female classification task, the top 10 genes in the aggregated panel using an RF evaluation model produced slightly poorer scores of 0.914 (GSE236932: [0.563, 1.0], GSE188715 [1.0, 1.0] and 0.878 (GSE236932: [0.571, 0.952], GSE188715: [0.857, 1.0]) for AUROC and F1 (0.863 composite), with a notably larger variability in performance across bootstrap samples for GSE236932. A notable decline in performance was observed in the male tumor *versus* female tumor task, with the aggregated top 25 genes yielding a composite score of 0.784. However, the average PR-AUC of 0.912 (GSE236932: [0.783, 0.982], GSE188715: [0.839, 0.983]) showed some degree of generalizability to unseen data.

**TABLE 2:** Best performing aggregate panels with n ≤ 50 for each classification task. For each aggregate panel, the number of genes, evaluation model, and average performance metrics across both external datasets are provided. In parentheses the 95% percentile bootstrap confidence intervals for GSE236932 and GSE188715, respectively, are provided. Degenerate confidence intervals ([1.0, 1.0]) were observed in GSE188715 for female AUROC and PR-AUC due to sample size limitations. The composite score was weighted to favor metrics better for imbalanced datasets: 0.5*F1 + 0.3*AUROC + 0.2*balanced accuracy.

Task	Top N	Model	Balanced accuracy	F1	AUROC	PR-AUC	Composite
Male tumor vs. non-tumor	50	Random forest	0.896 ([0.791, 0.967], [0.792, 0.977])	0.917 ([0.796, 0.967], [0.866, 0.978])	0.923 ([0.782, 1.0], [0.843, 0.979])	0.95 ([0.775, 1.000], [0.958, 0.996])	0.915
Female tumor vs. non-tumor	10	Random forest	0.750 ([0.571, 0.929], [0.5, 1.0])	0.878 ([0.571, 0.952], [0.857, 1.0])	0.914 ([0.563, 1.0], [1.0, 1.0])	0.863 ([0.460, 1.0], [1.0, 1.0])	0.863
Male tumor vs. female tumor	25	SVM	0.689 ([0.494, 0.862], [0.527, 0.858])	0.853 ([0.650, 0.907], [0.835, 0.959])	0.734 ([0.502, 0.909], [0.555, 0.913])	0.912 ([0.783, 0.982], [0.839, 0.983])	0.784

External validation was also carried out for the gene panels generated from ComBat-seq-merged data as opposed to z-score merging (Supplementary Tables S10 and S11). The aggregated male top 50 with a KNN evaluation model had an average F1 score of 0.914 (GSE236932: [0.811, 0.982], GSE188715: [0.854, 0.958]) and an AUROC of 0.945 (GSE236932 [0.915, 1.0], GSE188715: [0.848, 0.969]) with a composite score of 0.923. Furthermore, for females the aggregated top 10 panel with a SVM evaluation model attained an average F1 score of 0.949 (GSE236932: [0.792, 1.0], GSE188715: [0.5, 1.0]) and an average AUROC of 0.961 (GSE236932: [0.739, 1.0], GSE188715: [1.0, 1.0]), combining for a composite metric of 0.931. In general, the ComBat-seq-based panels performed better than those generated with z-score-merged data metrics and produced smaller confidence intervals across most metrics, especially in GSE236932.

### Pathway enrichment, PPI network analysis, and literature search

**FIGURE 7:**
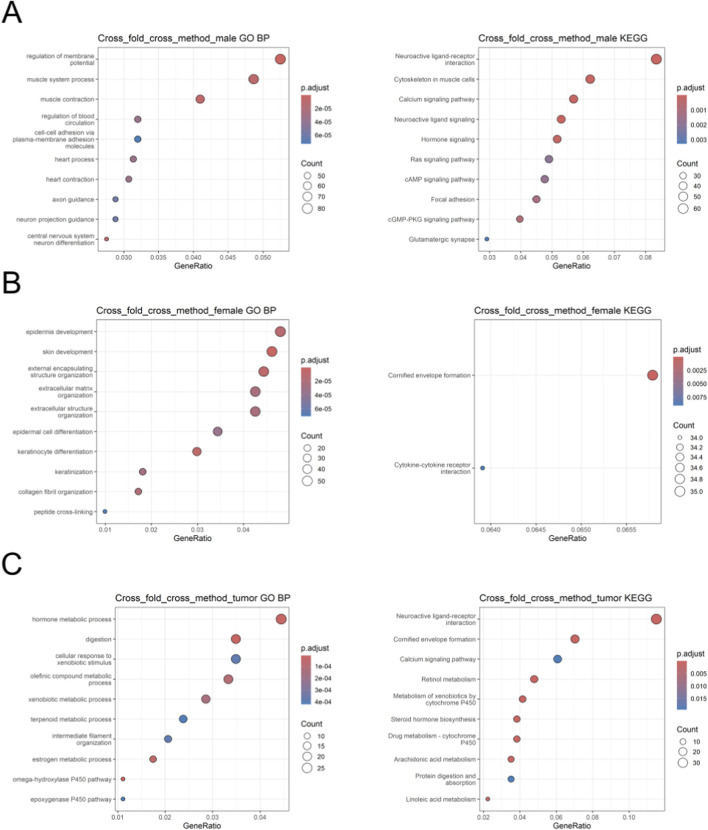
Dot plots for pathway enrichment analysis. Top 10 enriched terms for the GO biological process database in terms of q-value and gene ratio (# of genes associated with pathway: # of genes) and Top 10 enriched terms for the KEGG pathway database for the male **(A)**, female **(B)**, and tumor **(C)** cohorts. Note that the male and female tumor *versus* non-tumor top 500 genes yielded less than 10 significant enrichments for the KEGG database.

To determine the pathways and biological processes relevant to the machine learning-selected genes, enrichment analysis with GO and KEGG databases was performed (Figure 7). Only terms with a q-value of less than 0.05 were considered. When the aggregated top 300 panels were analyzed, only the male tumor vs. female tumor task yielded significant enrichments. The top ontologies were retinol metabolism and hormone metabolic process for KEGG pathways and GO biological process, respectively (Figure 7C). Relevant KEGG terms included the metabolism of drugs and xenobiotics by cytochrome P450, chemical carcinogenesis, and steroid hormone biosynthesis. In terms of GO biological process enrichments, xenobiotic response and metabolism, estrogen metabolic process, and cellular glucuronidation were especially pertinent. In order to get enrichments for the sex-specific panels, the search was expanded to include the top 500 genes of the aggregated panel. For male tumor *versus* non-tumor, the top terms were cGMP-PKG signaling and cell-substrate adhesion for KEGG and GO, respectively (Figure 7A). Other tumor-related ontologies included cytoskeleton in muscle cells, focal adhesion, hormone catabolic process, and junction assembly. For female tumors *versus* non-tumor, the terms with the lowest q-value, oddly, were cornified envelope formation and skin development for KEGG and GO (Figure 7B). However, some terms more relevant to the tumor context also appeared, like extracellular matrix organization, collagen fibril organization, and protease inhibition. When pathway analysis was attempted for the ComBat-seq-generated gene panels, no significant enrichments were obtained for the male-specific aggregated panel even when including all possible genes.

**FIGURE 8:**
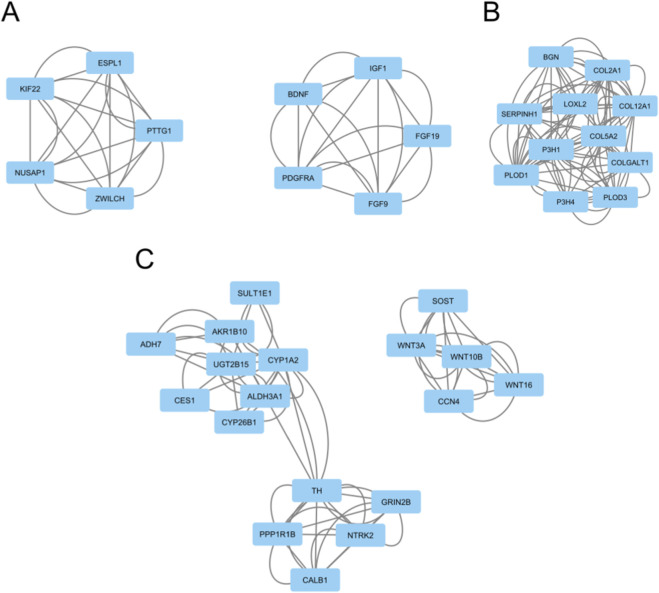
Significant gene modules containing hub genes in PPI network analysis for male **(A)**, female **(B)**, and tumor **(C)** cohorts. Hub genes were defined as genes in the top 10 maximum clique centrality ranking while significant gene modules were defined as having a score of 3.00 or greater.

A PPI network was also constructed for each of the aggregated gene panels corresponding to the three classification tasks. In the male panel, *ESPL1*, *NUSAP1*, *KIF22*, *ZWILCH*, *IGF1*, *PTTG1*, *PDGFRA*, *FGF9*, *MCM7*, and *DKC1* were the top 10 hub genes based on maximum clique centrality. Two significant gene clusters contained hub genes (Figure 8A). The first contained *PTTG1*, *KIF22*, *ZWILCH*, *NUSAP1*, and *ESPL1* and was related to sister chromatid separation and mitotic anaphase based on Reactome pathway enrichment. The second, comprised of *PDGFRA*, *BDNF*, *FGF19*, *IGF1*, and *FGF9*, was associated with aberrant PI3K-AKT signaling cancer. For the female cohort, *COL5A2*, *PLOD1*, *COL12A1*, *P3H1*, *COL2A1*, *BGN*, *SERPINH1*, *COLGALT1*, *PLOD3*, and *P3H4* were hub genes. One significant gene cluster contained all female hub genes, and was significantly enriched for collagen formation and extracellular matrix organization (Figure 8B). Finally, the hub genes for the male tumor *versus* female tumor network were *NTRK2*, *CALB1*, *UGT2B15*, *TH*, *CYP1A2*, *GRIN2B*, *ADH7*, *WNT3A*, *WNT10B*, and *WNT16*. Three significant gene modules contained hub genes (Figure 8C). The first cluster was made up of *CALB1*, *PPP1R1B*, *TH*, *GRIN2B*, and *NTRK2*, and was connected to NTRK signaling. The second, containing *WNT10B*, *SOST*, *CCN4*, *WNT3A*, and *WNT16*, was primarily related to WNT signaling and ligand biogenesis. The third cluster was the largest and consisted of *AKR1B10*, *SULT1E1*, *CYP1A2*, *UGT2B15*, *ALDH3A1*, *CES1*, *CYP26B1*, and *ADH7*. The enrichments for this final cluster were vague, with biological oxidations and phase I/II of metabolism.

Due to the difficulties in obtaining relevant enrichments, a detailed literature search was also performed for the top 20 genes in each aggregated panel (Table 3). Genes were categorized as BCa-related, tumor-related, or other. BCa-related genes had to be connected to BCa either through bioinformatics analyses or *in vitro*/vivo experiments, while tumor-related genes could be for any tissue type. Both the sex-specific development panels contained more BCa-relevant genes compared to the sex-related progression selection. The latter contained many more poorly characterized lncRNAs. In the male-specific panel, *PRAC1* was of particular interest due to its high ranking and connection to prostate cancer. Additionally, sex chromosome-linked genes like *PCDH11Y*, *TTTY10*, and *IL1RAPL1* appeared as strong predictors of male tumor development. On the female side, many genes related to androgen signaling, like *AR*, *USP54*, *PMEPA1*, and *PLXNA1* were in the top 20 of the aggregated panel. A separate literature search was also conducted for the ComBat-seq gene panels (Supplementary File 1). Overall, far fewer of the top 20 for each gender were linked to sex hormones and chromosomes and related to BCa. Due to this and the lack of significant enrichments for males, the z-score merged results were deemed more biologically relevant than what was obtained with ComBat-seq.

**TABLE 3:** Literature search of genes in the top 20 of the aggregated panel based on RRA score for each classification task. BCa-related genes appeared in either published bioinformatics analyses or *in vitro*/vivo experiments of BCa. Many of these were also relevant to other tumor types.

Task	BCa-related	Tumor-related	Other
Male Tumor vs. Non-Tumor	NDNF (Mo et al., 2021), PRAC1 (Kim et al., 2015; Mo et al., 2021; Xu et al., 2020), TOX2 (Jin et al., 2018), TRPA1 (Cucu, 2024), SNORD114-3 (He et al., 2020a), MTTP (Wang et al., 2025b), CCDC141 (Yang et al., 2021), MIR222 (Han et al., 2019)	PCDH11Y (Terry et al., 2006), TTTY10 (Chen et al., 2019; Zhang and Chen, 2022; Zhao et al., 2024; Zhu et al., 2017), IL1RAPL1 (Frenay et al., 2022), MIR125B1 (Zeng et al., 2014), MASP1 (Yu et al., 2024; Xiao et al., 2025), TCP10L (Shen et al., 2020), EYA1 (Nelson et al., 2024; Zhou et al., 2017), FCN2 (Wang et al., 2022a)	MIR4506, LOC101928516, MGARP, VCX2
Female Tumor vs. Non-Tumor	P3H1 (Zhang et al., 2023), CHPF (Zhong et al., 2023), AR (Miyamoto et al., 2012; Sikic et al., 2019), FAP (Mezheyeuski et al., 2020; Muilwijk et al., 2024), P3H4 (Liu et al., 2024; Hao et al., 2020), PLOD3 (Chen et al., 2023), USP54 (Zhou et al., 2024), PRSS27 (Wang et al., 2023), ANKRD37 (Shabbir et al., 2025), PMEPA1 (Qiu et al., 2021), INHBA (Lee et al., 2015)	ARSF (He et al., 2020b), PLXNA1 (Hu et al., 2024), COMP (Ma et al., 2022), FRMD3 (Fujino et al., 2025), GABRA4 (Yan et al., 2020), SLC38A7 (Haratake et al., 2021), TMEM217 (Yao et al., 2023), JPH4 (Chung et al., 2009)	LOC102724768
Male Tumor vs. Female Tumor	SNCB (Wang et al., 2022b), SLC7A11 (Shabbir et al., 2025; Shen et al., 2022), SLC2A14 (Shabbir et al., 2025)	SIGLEC14 (Zhang et al., 2021), SALL1 (Misawa et al., 2018; Ma et al., 2018), LOC153910 (Al Sharie et al., 2024), PROK2 (Lattanzi et al., 2022; Kurebayashi et al., 2015), TMSB15A (Darb-Esfahani et al., 2012), LINC01121 (Wang et al., 2019), LINC00567 (Wang et al., 2021), CES1 (Jiang et al., 2024; Ke et al., 2020), LINC02057 (Lei et al., 2025; Ma et al., 2023), NKAIN1 (Leyten et al., 2015), ACHE (Pérez-Aguilar et al., 2023), ARSF (He et al., 2020b), TTPA (Wright et al., 2009), LINC02432 (Tan et al., 2022)	LINC03020, LOC105375614, HEY2-AS1

## Discussion

This study adopts a novel approach in identifying sex-related BCa genes and highlights new candidate biomarkers for further experimentation. A recent study by Wang and colleagues highlighted sex-specific genes through DGEA, PPI network construction, pathway enrichment analysis, and immune cell infiltration correlation (Wang Y. et al., 2025). Here, DGEA was the starting point for a rigorous cross-validated machine learning workflow using four different feature selection techniques. Distinct sets of genes were generated by each selection method, therefore an ensemble-like approach with RRA was employed to generate robust, high-performing biomarker panels. In external validation, male and female-specific gene panels aggregated across fold schemes and feature selection techniques limited to 50 or fewer genes obtained average AUROC scores of 0.932 and 0.914, respectively. These models achieving AUROC values greater than 0.90 indicate a respectable diagnostic performance in the context of clinical predictions (Çorbacıoğlu and Aksel, 2023). Pathway enrichment and PPI analysis confirmed the molecular pathways associated with carcinogenesis in each sex, with males displaying a cell interaction, mitotic regulation, and PI3K-AKT-focused signature while the female panel consisted of extracellular matrix (ECM) organization and collagen synthesis-connected genes. A literature search of the genes in each sex-specific BCa development panel revealed that 4-5 top predictors were tumor-related and either sex-linked or associated with sex hormone signaling in both. In contrast, the male *versus* female tumor classification task displayed much poorer performance in both internal and external validation. Even in the best-performing aggregated panel, an average composite score of 0.784 with an average AUROC of 0.734 was obtained in external validation. However, a literature search showed that many of the top genes were still relevant to tumors and further analysis found that genes involved in xenobiotic metabolism were prominent in the aggregated panel.

For the male-specific tumor development genes, *PRAC1*, *TTTY10*, *PCDH11Y*, and *IL1RAPL1* were connected to sex-related chromosomes or molecular mechanisms. Alternative names for *PRAC1* include prostate cancer susceptibility candidate protein 1 and prostate, rectum and colon expressed gene protein. Previous studies have found that *PRAC1* loss in prostate cancer led to cell growth even in AR signaling inhibitor-treated samples and may act as a co-regulator of AR activity (Low et al., 2023). In the context of BCa, *PRAC1* methylation was shown to be significantly higher in BCa tissue and associated with higher tumor grade, as well as demonstrating significant downregulation in bioinformatics analyses (Kim et al., 2015; Mo et al., 2021; Xu et al., 2020). Interestingly, *PCDH11Y*, a Y-linked protein-coding gene, was identified as having increased mRNA expression in androgen-resistant prostate cancer cell lines (Terry et al., 2006). *TTTY10*, a Y-linked lncRNA, has been implicated in numerous tumor types, including colorectal cancer, papillary thyroid carcinoma, osteosarcoma, and esophageal squamous cell carcinoma (Chen et al., 2019; Zhang and Chen, 2022; Zhao et al., 2024; Zhu et al., 2017). Finally, *IL1RAPL1* is an X-linked gene thought to be involved in the pro-inflammatory response through NF-kB and AP-1 pathways that is overexpressed in tumors like pancreatic ductal adenocarcinoma, Ewing sarcoma, and triple-negative breast cancer (Frenay et al., 2022). Outside of genes connected to sex-specific chromosomes or pathologies, *MIR222* was also in the top 20 of the aggregated panel. Functional analyses have identified that *METTL3* promotes BCa proliferation by maturing pri-*MIR221/222*, which then go on to target *PTEN* (Han et al., 2019). Other genes like *NDNF*, *TOX2*, *TRPA1*, *SNORD114-3*, and *MTTP* have been noted as significantly downregulated, upregulated, and/or associated with pathological features in BCa compared to controls (Mo et al., 2021; Cucu, 2024; He et al., 2020a; Jin et al., 2018; Wang W. et al., 2025). Moreover, when performing pathway analysis on the top 500 genes in the aggregated panel, numerous tumor-relevant terms were significantly enriched. Wnt/β-catenin transcription has been linked to cGMP-PKG signaling (the top KEGG enrichment), leading to cancer cell expansion and immune evasion (Piazza et al., 2020). Specifically, in epithelial ovarian cancer, PKG activity was shown to alter EGF-elicited cell proliferation and migration (Lan et al., 2023). Moreover, focal adhesions are known to be connected to the tumor microenvironment, acting as the physical support connecting tumor cells to the surrounding ECM and allowing for mechanical signaling (Liu et al., 2025). Another significantly enriched term regarding cell interactions was junction assembly. The epithelial-to-mesenchymal transition, a prominent cancer hallmark, is known to disrupt the adhesive and signaling functionalities of many types of cell junctions (Knights et al., 2012). Moreover, PPI network analysis revealed hub gene-containing clusters associated with mitotic anaphase and PI3K-AKT signaling. Research has found that the genes responsible for sister chromatid adhesion are often underregulated in cancer, leading to chromosomal instability and poorer patient outcomes (Leylek et al., 2020). The top ranked hub gene in the mitosis-related module and entire male-specific PPI network, *ESPL1*, was found to be involved in enhanced cisplatin resistance *via* the JAK2/STAT3 pathway in BCa tissues and cells (Zhang et al., 2024). Hyperactive PI3K-AKT signaling is attributed to numerous cancer types, but in BCa it has been associated with the epithelial-to-mesenchymal transition specifically (Chi et al., 2022). While multiple key predictors in the male-specific aggregated panel were associated with androgen signaling and sex-linked tumor development, as a whole, the panel had strong themes of cell interactions, mitotic dysfunction, and aberrant PI3K-AKT signaling.

Looking at female-specific BCa development, *AR*, *PLXNA1*, *USP54*, *PMEPA1*, and *ARSF* were of particular interest. *AR* is often downregulated in BCa, and lower rates of AR positivity have been reported in high-grade cases compared to lower grades (Miyamoto et al., 2012). Furthermore, AR is known to be an independent prognosticator in females, but not in males (Sikic et al., 2019). *PLXNA1* has not been connected to BCa in the literature, but has been shown to alter AR inhibitor effectiveness in prostate cancer, with elevated expression enabling tumor proliferation even under enzalutamide treatment conditions (Hu et al., 2024). Additionally, *USP54* has displayed higher expression in prostate cancer and was correlated with AR signaling levels as well as heightened proliferation compared to silenced models (Zhou et al., 2024). *PMEPA1* (prostate transmembrane protein, androgen induced 1) silencing has been linked to significantly decreased BCa proliferation, migration, and invasion *in vitro* (Qiu et al., 2021). These genes may be related to aberrant androgen signaling leading to tumorigenesis in female BCa. Finally, *ARSF* is an X-linked gene that has not been connected to BCa, but was associated with overall survival in glioblastoma in one study (He et al., 2020b). In terms of significantly enriched pathways for the entire female-specific aggregated panel, several were related to extracellular matrix and collagen fibril organization. Compositional changes in the ECM are characteristic of tumor development, with tumors exhibiting greater stiffness due to upregulated collagen deposition by cancer-associated fibroblasts (CAFs) (Huang et al., 2021). Tumor-associated macrophages and CAFs in tandem influence collagen remodeling to produce a shift in fibril orientation, resulting in heightened cancer cell migration (Necula et al., 2022). This was supported by PPI network analysis as well, where the significant gene cluster containing all hub genes was found to be related to collagen formation and biosynthesis. The top hub gene among the female-specific genes was *COL5A2*. In bioinformatics analyses, BCa patients with lower *COL5A2* expression had better outcomes for tumor grade, migration, and overall survival (Zeng et al., 2018). However, unlike the male pathway enrichment, several seemingly unrelated ontologies, like cornified envelope formation and epidermis development, were highly enriched.

While the sex-related tumor progression classification demonstrated worse predictive performance, many of the top predictors were still related to BCa or other tumor types. *ARSF* and *TMSB15A*, both X-linked genes, were in the top 20 of the aggregated gene panel. As previously mentioned, *ARSF* is linked to tumor progression in other tumor types but has yet to be connected to BCa. On the other hand, *TMSB15A* has been linked to breast cancer treatment response (Darb-Esfahani et al., 2012). Several other non-sex-linked predictors were related to tumor-induced shifts in expression or cell behavior in BCa. Two solute carrier family genes, *SLC7A11* and *SLC2A14*, have demonstrated aberrant expression in BCa (Shabbir et al., 2025). Specifically, *SLC7A11* upregulation inhibited ferroptosis and promoted BCa progression in an *in vitro*/vivo study (Shen et al., 2022). Significant enrichments for the aggregated male tumor *versus* female tumor panel were centered around steroid hormones and xenobiotic metabolism/response. Recent research has shown that certain variants of xenobiotic metabolism genes are associated with higher-grade tumors or recurrence in BCa (da Silva et al., 2025). Furthermore, hormone metabolic process was among the top 10 most highly enriched GO biological process terms, but estrogen metabolic process specifically also had a q-value less than 0.05. In numerous *in vitro* studies, estrogens have proven to accelerate cell proliferation, potentially through phospho-ERK expression, as well as downregulate tumor suppressor genes in BCa cells (Goto and Miyamoto, 2021). Additionally, network analysis revealed a significant gene module associated with WNT signaling. WNT signaling has been implicated in multiple tumor types including BCa due to its activation of PI3K-AKT signaling, which then induces therapeutic resistance and a proliferative phenotype (Garg and Maurya, 2019). Indeed, associations between AR signaling and activity of ERK, WNT, or AKT in BCa cells have been suggested (Zheng et al., 2011; Li et al., 2013).

These results diverge from what Wang and company recently discovered through their analyses. Wang’s study identified the AR signaling pathway as enriched in male-specific hub genes through PPI network analysis, but here, AR expression was a strong predictor of female-specific BCa development (Wang Y. et al., 2025). This is in line with the work of Sikic and colleagues identifying the gene as an independent prognosticator in females (Sikic et al., 2019). Instead, Wang and coworkers found the Wnt signaling pathway to be highly enriched in female-specific hub genes. Out of the 300 genes in the machine learning-generated male-specific BCa development panel, only 6 genes overlapped with the male-specific hub genes identified in the Wang paper: *DDX11*, *PALB2*, *ESPL1*, *CDH17*, *PDGFRA*, and *TGFBR2*. No overlap was observed in the female-specific genes between this study and theirs. However, this alternative approach treating biomarker discovery as a feature selection problem still produced high-performance, biologically relevant findings. The majority of the top predictors in both male and female-specific aggregated panels were related to BCa or other tumor types. Genes with a heavy influence on male tumor classification across folds like *PRAC1*, *PCDH11Y*, *TTTY10*, and *IL1RAPL1* were linked to androgen signaling in other tumor types or associated with sex-linked chromosomes. In the female analysis, *AR*, *PLXNA1*, *USP54*, *PMEPA1*, and *ARSF* were still implicated in androgen activity, but suggest that different downstream pathways are affected compared to males. Pathway enrichment of both aggregated panels found that male-specific genes were related to cell adhesion and junctions while female-specific genes were more relevant to ECM and collagen organization. Finally, taking the respectable performance of the aggregated male and female-specific BCa development gene panels on external datasets into account, the aforementioned sex-related predictor genes identified in this study could be promising candidates for further experimentation *in vitro*/vivo.

This study has limitations to take into account as well. Due to the established difference in BCa occurrence between sexes, the datasets used for this analysis had limited female samples and therefore exhibited class imbalance. This was addressed through the use of a hybrid scoring system comprised of metrics sensitive to imbalanced datasets for hyperparameter optimization and feature selection. However, the small number of female samples still manifested through the large confidence intervals in performance metrics compared to males for external validation. Moreover, there was a notable drop in performance when going from the sex-specific BCa development panels to the sex-related BCa progression panel for both internal and external validation. Therefore, more weight was assigned to the sex-specific biomarkers in terms of their potential for further experimentation. More complex models integrating mutation or clinical data in addition to gene expression may be necessary to understand biomarkers of sex-related BCa progression. Finally, while the approach has been adopted in several machine learning-based studies of gene expression in cancer, independent z-scoring may not fully address the batch effects between different datasets and sequencing technologies. Still, the models trained on the merged dataset generalized well to external datasets after they had also been z-scored independently. Despite this, alternative normalization of the external datasets could shift performance, so the results obtained in this study should be considered research-grade rather than deployable. Using ComBat-seq as a merging technique offered an improvement in performance metrics and a narrowing of confidence intervals but produced far less genes relevant to sex-specific cancer biology. With sex-related biomarker discovery being the primary objective of this study, the z-score-merged results were favored for their increased biological relevance.

## Data Availability

The scripts used to generate the results in this study can be found on GitHub: https://github.com/rit-cui-lab/Machine-learning-based-determination-of-sex-related-bladder-cancer-biomarkers. The TCGA-BLCA (https://portal.gdc.cancer.gov/projects/TCGA-BLCA), GTEx (https://gtexportal.org/home/downloads/adult-gtex/bulk_tissue_expression), GSE133624 (https://www.ncbi.nlm.nih.gov/geo/query/acc.cgi?acc=GSE133624), GSE236932 (https://www.ncbi.nlm.nih.gov/geo/query/acc.cgi?acc=GSE236932), and GSE188715 (https://www.ncbi.nlm.nih.gov/geo/query/acc.cgi?acc=GSE188715) datasets are also available online.
